# A Comparative Study of Acute Alcoholic Hepatitis vs. Non-Alcoholic Hepatitis Patients from a Cohort with Chronic Alcohol Dependence

**DOI:** 10.3390/genes14040780

**Published:** 2023-03-23

**Authors:** Kyaw Min Tun, Zahra Dossaji, Blaine L. Massey, Kavita Batra, Chun-Han Lo, Yassin Naga, Salman Mohammed, Abebe Muraga, Ahmad Gill, Dwaipayan Mukhopadhyay, Ashok Singh, Daisy Lankarani, Jose Aponte-Pieras, Gordon Ohning

**Affiliations:** 1 Department of Internal Medicine, Kirk Kerkorian School of Medicine at UNLV, University of Nevada, Las Vegas, NV 89102, USA; 2 Department of Medical Education, Kirk Kerkorian School of Medicine at UNLV, University of Nevada, Las Vegas, NV 89102, USA; 3 Office of Research, Kirk Kerkorian School of Medicine at UNLV, University of Nevada, Las Vegas, NV 89102, USA; 4 Department of Internal Medicine, University of South Florida Morsani College of Medicine, Tampa, FL 33612, USA; 5 Kirk Kerkorian School of Medicine at UNLV, University of Nevada, Las Vegas, NV 89102, USA; 6 Department of Mathematical Sciences, University of Nevada, Las Vegas, NV 89154, USA; 7 Department of Resorts, Gaming & Golf Management, University of Nevada, Las Vegas, NV 89154, USA; 8 Insite Digestive Health Care, Tarzana, CA 91356, USA; 9 Division of Gastroenterology and Hepatology, Department of Internal Medicine, Kirk Kerkorian School of Medicine at UNLV, University of Nevada, Las Vegas, NV 89102, USA

**Keywords:** alcohol use, alcoholic hepatitis, prevention

## Abstract

The rate of alcoholic hepatitis (AH) has risen in recent years. AH can cause as much as 40–50% mortality in severe cases. Successful abstinence has been the only therapy associated with long-term survival in patients with AH. Thus, it is crucial to be able to identify at-risk individuals in order to implement preventative measures. From the patient database, adult patients (age 18 and above) with AH were identified using the ICD-10 classification from November 2017 to October 2019. Liver biopsies are not routinely performed at our institution. Therefore, patients were diagnosed with AH based on clinical parameters and were divided into “probable” and “possible” AH. Logistic regression analysis was performed to determine risk factors associated with AH. A sub-analysis was performed to determine variables associated with mortality in AH patients. Among the 192 patients with alcohol dependence, there were 100 patients with AH and 92 patients without AH. The mean age was 49.3 years in the AH cohort, compared to 54.5 years in the non-AH cohort. Binge drinking (OR 2.698; 95% CI 1.079, 6.745; *p* = 0.03), heavy drinking (OR 3.169; 95% CI 1.348, 7.452; *p* = 0.01), and the presence of cirrhosis (OR 3.392; 95% CI 1.306, 8.811; *p* = 0.01) were identified as characteristics more commonly found in the AH cohort. Further, a higher inpatient mortality was seen in those with a probable AH diagnosis (OR 6.79; 95% CI 1.38, 44.9; *p* = 0.03) and hypertension (OR 6.51; 95% CI 9.49, 35.7; *p* = 0.02). A higher incidence of mortality was also noted among the non-Caucasian race (OR 2.72; 95% CI 4.92; 22.3; *p* = 0.29). A higher mortality rate despite a lower incidence of alcohol use among non-Caucasian patients may indicate healthcare disparities.

## 1. Introduction

Alcohol use is responsible globally for approximately 3 million deaths per year, accounting for 5.3% of all deaths, according to a 2022 report from the World Health Organization (WHO) [1]. Excessive alcohol use is also the third leading cause of preventable deaths in the United States (U.S.) and contributes to the development of acute alcoholic hepatitis (AH) [2]. Furthermore, it has been estimated that 10 to 15% of patients in the U.S. who chronically consume alcohol develop alcohol-associated liver disease (ALD) [2]. AH is a manifestation of ALD, a spectrum of liver injury that begins with steatosis and can potentially progress to acute alcoholic hepatitis, alcohol-associated cirrhosis, and AH with acute or acute-on-chronic liver failure [3].

AH is a clinical syndrome with a hallmark presentation that includes rapid onset of jaundice, hepatomegaly, ascites, encephalopathy, and generalized signs or symptoms including fever, abdominal pain, or muscle wasting [3,4]. However, individuals with AH may also present with only mild symptoms or non-specific laboratory abnormalities. Therefore, determining the incidence of AH can be an obstacle in part due to diagnostic challenges. Other factors, such as comorbidities and improper ICD (international classification of diseases) coding, can further undermine the accuracy of the AH incidence rate [3].

Nonetheless, the rate of AH has risen in recent years, particularly in the relatively younger population with an average age of 53 years [3,4]. In patients with severe AH, which can be determined by a Maddrey’s discriminant function (MDF) value greater than 32, six-month mortality can be as high as 40% [5]. Previously reported risk factors for AH among individuals with alcohol use include female gender, high body mass index, genetic susceptibility, malnutrition, tobacco dependence, and concomitant liver diseases [2,3,5]. Liver biopsy in patients with AH displays distinct histopathological patterns consistent with hepatocellular injury, such as lobular inflammation, hepatocyte ballooning, micro- and macro-vesicular steatosis, and fibrosis [3]. While “definite” diagnosis of AH requires liver biopsy, AH can be clinically diagnosed and categorized as “probable” if there are no confounding factors or “possible” if there are potential confounding factors [3]. If the clinical and laboratory criteria are not met and/or if there is an alternative explanation for a patient’s presentation, the patient is categorized as non-AH.

Once diagnosed, there are several stratification algorithms, such as MDF, used to predict disease severity and mortality. Successful abstinence from alcohol has been the only intervention or therapy associated with long-term survival in patients with AH [3,4,5,6]. Therefore, it is critical to identify at-risk individuals in order to provide personalized counseling on alcohol use disorder and implement preventative measures.

A retrospective comparative analytical study was performed to identify the risk factors associated with developing AH among patients with chronic alcohol dependence at a single tertiary institution located in the metropolitan area of Las Vegas, Nevada.

## 2. Methods

### 2.1. Study Design

We performed a retrospective review of patient charts from the medical record database at a single tertiary academic county medical institution. The study, along with the waiver of informed consent, was approved by the institutional review board (IRB) at the University Medical Center of Southern Nevada (UMC), Las Vegas, Nevada (IRB number: UMC-2019-248, approved 10 November 2021). We first queried the UMC electronic medical record database using the following criteria: adult patients (age 18 and older) who were admitted for elevated transaminases, elevated bilirubin, alcohol-induced liver injury including alcoholic hepatitis, or alcohol use disorder were identified using the ICD-10 classification (International Classification of Diseases, tenth revision) over a 35-month period extending from 1 November 2017 to 10 October 2019. We did not extend the study beyond October 2019 as to avoid additional confounding factors and elevated aminotransferases that can occur with the disease due to the 2019 coronavirus (COVID-19).

### 2.2. Selection Criteria and Measures

While there is a spectrum of diagnostic criteria for alcohol use disorder based on the Diagnostic and Statistical Manual of Mental Disorders, Fifth Edition [7], patients were included in our study on initial screening if there was a diagnosis of alcohol use disorder on medical coding or reported use of alcohol for at least 6 months with less than 60 days of abstinence [3]. Afterwards, additional data was retrieved regarding the pattern of alcohol use. A patient was determined to be a binge drinker if there was consumption of 5 or more standard drinks in males or 4 or more standard drinks in females in a 2-h period as defined by the National Institute on Alcohol Abuse and Alcoholism (NIAAA) [3,8]. A heavy drinking pattern was defined as consuming more than 4 standard drinks on any day, more than 14 standard drinks per week in males, more than 3 standard drinks on any day, or more than 7 standard drinks per week in females, in accordance with the definition from the NIAAA [8]. A standard drink in the United States is described by the NIAAA as containing 14 g of ethanol, as found in 5 fluid ounces of wine, 12 fluid ounces of beer, and 1.5 fluid ounces of distilled spirits such as vodka, hard liquor, and tequila [9,10]. The alcohol percentage in each drink was approximated at 5%, 12%, and 40% in beer, wine, and hard liquor/distilled spirits, respectively, based on prior literature standards [10].

Liver biopsies are not routinely performed on patients with suspected AH at our institution. Hence, the diagnosis of AH was made clinically, both from the ICD-10 coding and from a review of the charts by the authors. Patients were diagnosed with probable AH if all of the following criteria were met: onset of jaundice within the past 8 weeks; ongoing consumption of alcohol for 6 or more months with less than 60 days of abstinence before the onset of jaundice; an aspartate aminotransferase (AST)/alanine aminotransferase (ALT) ratio > 1.5 with both values < 400 IU/L; an AST > 50 IU/L; and a serum total bilirubin > 3.0 mg/dL [3]. If some but not all criteria were satisfied, or if there was the presence of potential confounding factors including but not limited to ischemic hepatitis, cocaine use, drug-induced liver disease, and metabolic liver disease, or if alcohol use could not be assessed properly based on chart review, the patient was allocated to a possible AH category [3]. Exclusion criteria were age younger than 18, abstinence from alcohol for ≥60 days, outpatient status, and a diagnosis of neither probable nor possible AH. If there were multiple hospitalizations for AH during the study period, only the latest encounter was included in our study. The AH cohort was also divided into two categories. Patients who were diagnosed with AH for the first time were labeled as first-time AH. On the other hand, patients who had previously had at least one documented episode of AH were classified as having recurrent AH. Using ICD-10 classification, a patient’s medical chart from several local hospitals was reviewed through an interconnected electronic health records system to determine whether the patient had a prior diagnosis of AH.

The hepatotoxicity profile of the home medications of the patients was assessed using a grading system from the database of the National Library of Medicine of the National Institutes of Health [11]. A 5-point scale was used to estimate the level of hepatotoxicity of a medication: A = well-known cause; B = highly likely cause; C = probable cause; D = possible cause; E = unlikely cause or suspected but unproven cause [11]. If a medication was from category A, B, C, or D, drug-induced liver injury was determined to be a confounding factor, and the patient was classified as “Possible AH” if he or she met the diagnostic criteria otherwise. If a patient was taking several hepatotoxic medications, a decision was made to include or exclude him from AH based on the history, clinical symptoms, and laboratory data available in the patient’s chart since the laboratory anomalies could also be induced by the medications.

### 2.3. Data Collection

The data was collected from patient charts between 1 November 2017 and 10 October 2019. The data was divided into three categories: sociodemographic and behavioral history, clinical or medical characteristics, and hospital outcomes. Sociodemographic and behavioral history data included age, body mass index (BMI), gender, race, health insurance status, homelessness, prior history of AH, family history of alcohol use, duration of alcohol use, drinking pattern, percentage of alcohol content in the type of drink reported, tobacco use, and illicit drug use, including intravenous (IV) drug use. Clinical or medical information included: presence of encephalopathy, cirrhosis, ascites, use of hepatotoxic medications, presence of viral hepatitis, model for end-stage liver disease-sodium score (MELD-Na) at admission, MDF score at admission, liver biopsy report if performed, hypertension, hyperlipidemia, glycated hemoglobin (HgbA1c), human immunodeficiency virus (HIV) status, and whether treatment with glucocorticoids was required during the hospitalization for AH. Hospital outcomes such as disposition, inpatient mortality, and length of hospital stay were also documented. During the data collection, patients with possible or probable AH were labeled as such in their respective categories and were also listed under the umbrella category of AH.

### 2.4. Sample Size Justification and Power Analysis

Power was ascertained separately for t, chi-square, and multiple logistic regression by using Cohen’s effect size conventions (effect size = 0.5 for t-tests; effect size = 0.3 for the chi-square test) [12,13,14]. For the logistic regression analysis, we utilized the formula proposed by Green et al. (146, N ≥ 50 + 8 m), where ‘m’ corresponds to the number of predictors. The total number of predictors was 12, according to which N = 146 was deemed appropriate [15]. The total sample size estimated with a power of 0.80 was 128 and 143 for the t-test and chi-square test, respectively. The sample size with the greatest value (N = 146) was considered appropriate since it satisfies the minimum requirement of all the statistical tests used.

### 2.5. Statistical Analysis

First, the data was recoded for running analytical operations. All assumptions, including normality and homogeneity of variance, were assessed. Categorical variables were represented as frequencies and proportions, whereas normally distributed continuous variables were represented by means and standard deviations. A square root transformation was applied to the non-normally distributed variables for the normal approximation. The Chi-square/Fisher exact test was used for comparing the categorical groups. Adjusted standardized residuals greater than 2 were considered significant cells for contingency tables larger than 2 × 2. Continuous outcomes among two groups (AH vs. non-AH, probable vs. possible AH) were compared using an independent-samples t-test or a Welch t-test. A multivariate logistic regression model was fit to generate adjusted odds ratios for the likelihood of alcoholic hepatitis as an outcome. Estimates of parameters were obtained through the maximum likelihood estimation method with 95% Wald’s confidence limits for the logistic model. The final model was selected based upon the Akaike Information Criterion (AIC) and the Schwarz Criterion (SC) [16]. Additional regression analyses were performed to generate an adjusted odds ratio for the likelihood of inpatient mortality as an outcome within the AH cohort.

For regression analyses, polytomous categorical variables were dummy coded to calculate accurate parameters. All tests were two-sided, and a *p*-value of <0.05 was considered significant. The Statistical Package for Social Sciences for Windows, version 27.0 (SPSS, Chicago, IL, USA), and Statistical Analysis System (SAS 9.4, Cary, NC, USA) were used to analyze the data for multivariate logistic regression.

## 3. Results

There were a total of 298 patients who were admitted to our tertiary teaching hospital in Southern Nevada from 1 November 2017 to 10 October 2019 and met the initial screening criteria. Of the 298 patients, 106 were determined to have no history of alcohol dependence and were subsequently excluded from the study. From the remaining cohort, 100 patients were diagnosed with AH and were listed under the AH cohort; 92 patients were determined not to have AH using the criteria mentioned previously and were categorized under the non-AH cohort. We performed a bivariate comparison of AH and non-AH patients in three categories: socio-demographic/behavioral history, clinical or medical characteristics, and hospital outcomes (Table 1, Table 2 and Table 3). Patients with AH were slightly younger, with a mean age of 49.3 years compared to that of non-AH patients at 54.5 years (*p* = 0.008). BMI and gender distribution were similar between the AH and non-AH cohorts. A higher incidence of AH was observed among non-Hispanic whites compared to other races (*p* = 0.02). Prior history of AH was correlated with a higher risk of developing AH (*p* = 0.007). Certain alcohol consumption patterns, such as binge drinking (*p* < 0.001), heavy drinking (*p* < 0.001), and the percentage of alcohol in the consumed beverage (*p* = 0.002), were also associated with AH. Other socio-economic factors such as health insurance status, homelessness, family history of alcohol use, tobacco use, and illicit drug use did not display a statistically significant correlation with AH (Table 1).

A higher incidence of AH was noted in patients with underlying liver diseases (*p* < 0.001), cirrhosis (*p* < 0.001), a high MELD-Na score (*p* < 0.001), or those who presented with ascites (*p* < 0.001). For AH patients, the mean MDF score was 22.1 ± 8.58 and the mean MELD-Na score was 20.9 ± 8.7 (Table 2).

The most frequently seen etiology of chronic liver disease in our sample was alcohol-related (N = 60 (60%) in the AH cohort and N = 25 (25.17%) in the non-AH cohort). The remaining medical characteristics, such as viral hepatitis, hypertension, or hyperlipidemia, did not display a statistically significant relationship with AH (Table 2). Out of the total 192 patients, only 70 (36.5%) had HgbA1c information available. Therefore, the presence or absence of diabetes/prediabetes could not be accurately determined for all patients from the sample and was not included in the analysis. There were only three records of concomitant positive viral hepatitis among the AH cohort; all three incidences were due to chronic Hepatitis C virus infection (HCV). In the non-AH cohort, there were 15 patients with a positive viral hepatitis panel. Thirteen patients were tested positive for HCV, one for chronic hepatitis B virus (HBV), and one for both HCV and chronic HBV. Lastly, the diagnosis of AH did not have a statistically significant impact on disposition, inpatient mortality, or length of hospital stay (Table 3).

Among the AH cohort, there were 43 patients (43%) with probable AH and 57 patients (57%) with possible AH. There were only 6 liver biopsies available; therefore, a definite diagnosis of AH could not be made in most patients and was not included as a sub-category. In addition, the AH cohort was also divided into first-time AH and recurrent AH. There were 71 patients (71%), who were diagnosed with AH for the first time, and 29 patients (29%), who had recurrent AH. In the latter group, there were 19 patients (65.52%), 4 patients (13.80%), 5 patients (17.24%), and 1 patient (3.45%) who, respectively, had one, two, three, and four episodes of AH prior to the index presentation.

**Figure 1:**
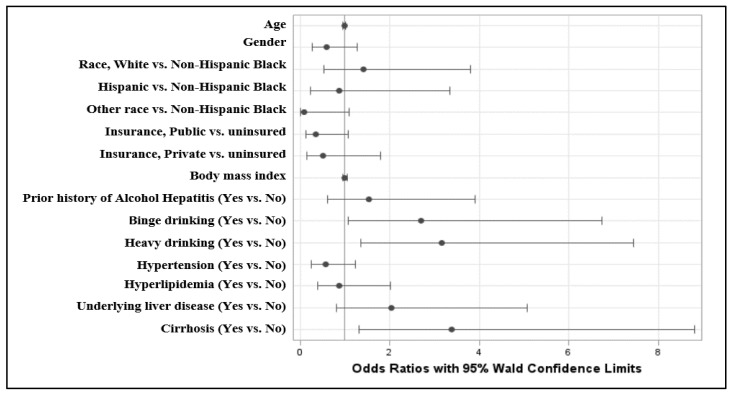
Forest plot showing odds ratio estimates for likelihood of Alcoholic Hepatitis.

A multivariate logistic regression analysis between the AH and non-AH cohorts was then performed on the overall cohort, using the development of AH as the outcome. Binge drinking was associated with a higher risk of developing AH (odds ratio [OR], 2.698; 95% confidence interval [CI], 1.079–6.745; *p* = 0.03), as was heavy drinking (OR, 3.169; 95% CI, 1.348–7.452; *p* = 0.01) (Table 4). The presence of cirrhosis was also associated with a greater likelihood of developing concurrent AH (OR, 3.392; 95% CI, 1.306–8.811; *p* = 0.01). The presence of cirrhosis also predisposes patients to AH (OR, 3.392; 95% CI, 1.306–8.811; *p* = 0.01). Other variables were not statistically significant. A forest plot with the OR estimates for the likelihood of AH with respect to particular variables is demonstrated in Figure 1.

A logistic regression analysis between probable and possible AH groups was also performed within the AH cohort, examining the outcome of inpatient mortality. The results are shown in Table 5. Patients with probable AH had a higher risk of inpatient mortality compared to those with possible AH (OR, 6.79; 95% CI, 1.38–44.9; *p* = 0.03). Concomitant hypertension was also associated with a higher probability of inpatient mortality amongst AH patients (OR, 6.51, 95%; CI, 1.49–35.7; *p* = 0.02).

## 4. Discussion

Alcoholic hepatitis falls under the spectrum of alcohol-associated liver diseases. The rate of alcohol consumption and incidence of AH, as well as binge and heavy drinking patterns, have been rising in the U.S. in the past few decades [17,18]. For instance, the proportion of patients born between 1945 and 1965 who were admitted to 169 medical centers in the U.S. with a primary diagnosis of AH increased from 26% to 31% from the year 2000 to 2011 [17]. Additionally, a study from 2003 reported that alcohol consumption was responsible for 44% of all deaths among liver disease patients [2].

Our study demonstrated that binge and heavy drinking lead to a higher risk of developing alcoholic hepatitis, which is consistent with prior studies [4,5,6]. The alcohol content within the consumed beverage is also a crucial variable. Our results were consistent with those from prior studies that showed heavy drinking is correlated with the development of AH [4,8,18,19].

Moreover, even a single episode of binge drinking can lead to increased levels of serum endotoxin (lipopolysaccharide) and 16S ribosomal DNA, which are markers of dysbiosis and translocation of the gut microbiome to the bloodstream [20]. The endotoxin subsequently causes increased levels of inflammatory markers, which can induce a dysregulated immune response that in turn increases the risk for AH [20].

However, only about 6 to 20% of individuals with a heavy drinking pattern develop AH [4]. Therefore, other risk factors such as gender, genetic predisposition, race, and type of beverage also contribute to the risk of developing AH. For example, although it was not noted in our study, it has been previously demonstrated that women can develop alcohol-related liver injury at lower levels of alcohol consumption [3,4,19]. A possible explanation offered for this relationship is the higher level of serum endotoxin in women compared to men during alcohol intake [20]. In comparison to non-Hispanic Whites, African Americans and Asian Americans/Pacific Islanders also have lower hospitalization rates due to AH, whereas higher hospitalization rates have been observed amongst Hispanics and Native Americans [21,22]. Similar results were seen in our study, where the majority of AH patients were non-Hispanic whites. Nevertheless, we discovered that non-Caucasian Americans have a higher rate of mortality compared to their Caucasian counterparts (OR 2.72; 95% CI: 0.492–22.3; *p* = 0.29). The higher mortality rate despite a lower rate of hospitalizations among the non-Caucasian American demographic may be indicative of disparities in healthcare access.

Our data revealed a higher incidence of AH in patients with cirrhosis than in their non-cirrhotic counterparts. This association may be explained by the impaired metabolism of alcohol due to defective hepatic function, leading to an increased buildup of lipopolysaccharide endotoxin and subsequent activation of inflammatory cytokines [20]. In addition, the presence of cirrhosis at the time of admission may suggest a prolonged history of alcohol use or frequent at-risk alcohol use patterns such as binge drinking or heavy drinking.

We identified probable AH and hypertension as two characteristics associated with inpatient mortality among our AH cohort. In patients with probable AH, patients display more binge or heavy drinking patterns and present with more severe laboratory abnormalities, signifying a higher degree of hepatic injury [3,18]. Hypertension has not been previously demonstrated in the literature to be related to inpatient mortality in alcoholic hepatitis. However, hypertension can sometimes be suggestive of underlying cardiovascular disease, and alcohol intake has been demonstrated to have a J- or U-shaped relationship with cardiovascular ailment, indicating that while an inverse correlation with total mortality is seen in individuals with light alcohol consumption (2–4 drinks per day for men and 1–2 drinks per day for women), excessive alcohol consumption may be associated with cardiovascular complications and mortality [23,24,25]. More specifically, two meta-analyses have concluded that hypertension is correlated with >20 g of alcohol intake per day in women and >30 g of alcohol intake per day in men [26,27]. This suggests that hypertension may be indicative of excessive alcohol use and precede subsequent cardiovascular damage via increased oxidative stress and imbalances in neurohormonal pathways [25]. Given the lack of longitudinal follow-up in our study, it was not possible to infer the relationship between alcohol intake and cardiovascular disease from our data.

Another principle that implicates hypertension in the development of liver disease is via the renin-angiotensin system (RAS) [28]. It has been established that the classical RAS axis produces angiotensin II, which can induce a pro-oxidant, pro-inflammatory, and fibrogenic effect on the liver [28]. Conversely, the counter-regulator RAS axis generates angiotensin 1–2, which negates the action of angiotensin II as an anti-oxidant and anti-fibrogenic agent [28]. Angiotensin-converting enzyme inhibitors or angiotensin receptor blockers inhibit the production of angiotensin II and have been shown to be beneficial in the treatment of chronic liver diseases. However, further clinical trials are required to determine their efficacy and safety profile in patients with alcoholic liver disease. Among the 100 patients with AH in our sample, there were 84 patients with underlying liver disease, of which hepatic steatosis was the most common (N = 71). We acknowledge that in patients with metabolic syndrome, it is not possible to distinguish between alcoholic-related liver disease and non-alcoholic fatty liver disease (NAFLD) even with a biopsy [3]. Hence, markers of the metabolic syndrome such as diabetes (A1c ≥ 6.5), dyslipidemia, and/or BMI ≥ 25 were included as confounding variables, and patients with such features were categorized as having a possible AH due to the degree of alcohol use. Thus, among 71 patients with hepatic steatosis, there were 43 patients with possible AH and 28 patients with probable AH. Only 3 patients, all of whom tested positive for the hepatitis C virus, were noted to have viral hepatitis data; therefore, a bivariate or logistic regression analysis could not be performed. Prior data in the literature has noted that patients with hepatitis C who have a heavy drinking pattern tend to develop a higher stage of fibrosis and viremia than their non-AH counterparts [4].

As a county catchment hospital, a large percentage of our patients were insured by public sectors such as Medicare and Medicaid; there were no insurance-specific differences noted.

Our study excluded patients starting from the beginning of the COVID-19 pandemic to minimize confounding variables. COVID-19 has been reported to cause varying degrees of liver enzyme abnormalities, which would have complicated data interpretation in our retrospective study when compared to patients in the pre-pandemic era [29,30]. Additionally, the pandemic has led to an overall increase in alcohol consumption and the incidence of AH. In a regional study [from Fresno, California] by Sohal et al., a 69% increase in AH-related hospitalization was noted after implementation of stay-at-home orders [31]. More specifically, there was a 100% increase in hospitalization of patients under 40 years old and a 125% increase in female patients; only a 34% rise was noted in males [31]. It is hypothesized that the younger individuals and females experienced a higher burden of economic, social, and psychological stressors from the pandemic, which led to increased alcohol use.

AH can cause as high as 40–50% mortality in severe cases, which is indicated by a MDF score > 32 [4]. Currently, abstinence from alcohol remains the sole management recommendation associated with long-term survival [3,4,18,19]. Treatment with prednisolone in severe cases of AH correlates with a reduction in 28-day mortality but not long-term survival [19]. Pentoxifylline, a phosphodiesterase inhibitor, is no longer used due to a lack of associated short-term or long-term survival benefit based on the data from the STOPAH trial (steroids or pentoxifylline for alcoholic hepatitis trial) [19]. There is yet no generalized consensus or validated effectiveness for other novel approaches, including vitamin E, N-acetylcysteine, anti-tumor necrosis factor-alpha, granulocyte-colony stimulating factor, and fecal microbiota transplantation [4,18,19].

Our study was conducted at a hospital that serves a metropolitan area with a high rate of alcohol consumption. Nevada is recognized as a state with one of the highest estimates of binge drinking, especially among individuals aged 18–34 [32]. Among the several risk factors for AH that have been validated in the literature, our study emphasizes certain patterns, such as binge drinking and heavy drinking, that may be prevalent in other similar metropolitan settings. The information from our study will allow healthcare professionals to customize their approach to addressing alcohol dependence in the community. Furthermore, our study demonstrated that probable AH and underlying hypertension are correlated with increased inpatient mortality. Consequently, a multidisciplinary approach can be designed in a timely fashion to implement preventative measures in patients at higher risk or from disadvantaged socioeconomic backgrounds.

We acknowledge several limitations in our study. First, although we have extensively adjusted for demographic, lifestyle, and clinical risk factors for AH, as with all observational studies, we cannot rule out the possibility of residual confounding. Second, our cohort was comprised of patients presenting to a single tertiary center; therefore, our findings may not be generalizable to milder AH patients. Third, alcohol intake was self-reported, leading to the possibility of recall bias as well as inaccurate quantification of alcohol intake, especially in those with an extensive history of alcohol consumption. The percentage of alcohol in the beverages consumed by the patients was estimated into three categories for the purpose of analysis, which could lead to overgeneralization. Fourth, since liver biopsies were not routinely performed for the diagnosis of AH at our institution, a diagnosis of “definite AH” could not be made in most patients. However, a 2020 practice guideline from the American Association for the Study of Liver Diseases states that AH can be diagnosed clinically based on history, presenting symptoms, and laboratory criteria [3]. Additionally, the use of biopsy in AH is usually limited to clinical trials, may not be routinely available in all clinical settings, and is further limited by inter-pathologist variability. Lastly, due to the lack of sufficient information available, a correlation between diabetes and AH, if any, could not be investigated.

## 5. Conclusions

Our study demonstrated that the incidence of AH was higher in younger patients. We also demonstrated that binge drinking, and/or heavy drinking correlate with the development of alcoholic hepatitis. A higher incidence of AH was also found in patients with cirrhosis. Hypertension and probable AH were also correlated with increased inpatient mortality. Higher mortality rates and a lower hospitalization rate among African American patients may be reflective of healthcare disparities in our metropolitan county hospital. Our results further suggest the presence of a strong relationship between cardiovascular disease and the inflammatory state induced by alcohol consumption. Abstinence remains the only treatment that can lead to long-term survival. The data from our study can be used to better identify patients at risk of developing alcoholic hepatitis. Interventions such as motivational counseling, timely guidance to community resources, and closer monitoring may be beneficial in this patient population. Further studies that investigate cardiovascular impairment and AH, such as whether adequate treatment of cardiovascular diseases lowers the risk of AH, may be warranted.

## Figures and Tables

**Table 1:** Bivariate comparisons of socio-demographic and behavioral history of the sample (N = 192).

Variable	Categories	AH, n (%) 100 (52.1)	Non-AH, n (%), 92 (47.9)	*p*-Value	Test Statistics	Effect Size
Age (Mean ± SD)	-	49.3 ± 12.0	54.5 ± 14.2	**0.008**	−2.701	−0.390
BMI (Mean ± SD)	-	27.9 ± 8.5	27.6 ± 8.1	0.7	0.288	0.042
Gender	Male	64 (64.0)	63 (68.5)	0.5	0.429	0.047
	Female	36 (36.0)	29 (31.5)			
Race	White	76 (76.0)	52 (56.5)	**0.02**	9.435	0.222
	Black	13 (13.0)	24 (26.1)			
	Hispanic	10 (10.0)	12 (13.0)			
	Other	1(1.0)	4 (4.3)			
Health insurance	Public	59 (59.0)	60 (65.2)	0.3	2.472	0.113
	Private	22 (22.0)	22 (23.9)			
	Uninsured	19 (19.0)	10 (10.9)			
Homelessness	Yes	17 (17.0)	18 (19.6)	0.6	0.212	0.033
	No	83 (83.0)	84 (80.4)			
Prior history of AH	Yes	29 (29.0)	12 (13.0)	**0.007**	7.264	0.195
	No	71 (71.0)	80 (87.0)			
Family history of alcohol use	Yes	10 (10.0)	5 (5.4)	0.2	1.387	0.085
	No	90 (90.0)	87 (94.6)			
Duration of alcohol use in years (Mean ± SD)	-	16.0 ± 11.6	18.5 ± 11.4	0.2	−1.292	−0.217
Binge drinking	Yes	51 (51.0)	15 (16.3)	**<0.001**	25.570	0.365
	No	49 (49.0)	77 (83.7)			
Heavy drinking	Yes	74 (74.0)	33 (35.9)	**<0.001**	28.238	0.383
	No	26 (26.0)	59 (64.1)			
% of Alcohol (Mean ± SD)	-	24.1 ± 17.0	16.6 ± 15.5	**0.002**	3.105	0.462
Tobacco use	Yes	47 (47.0)	53 (57.6)	0.1	2.161	0.106
	No	53 (53.0)	39 (42.4)			
Pack years (Mean ± SD)	-	21.5 ± 19.7	24.5 ± 24.7	0.5	−0.653	−0.132
IV drug use	Yes	6 (6.0)	5 (5.4)	0.9	0.028	0.012
	No	94 (94.0)	87 (94.6)			
Non-IV drug use	Yes	42 (42.0)	42 (45.7)	0.6	0.260	0.037
	No	58 (58.0)	50 (54.3)			

Notes: Other race includes Asian, Pacific Islanders, Native American and Alaska Native; Public insurance includes Medicare, Medicaid and VA; *p* values less than 0.05 are considered statistically significant and are bolded in the table. Data are represented as frequencies and proportions unless stated otherwise. Among those who had previous history of AH, number of episodes varied from 1 (min.) to 4 (max.).

**Table 2:** Bivariate comparisons of clinical or medical characteristics of the sample (N = 192).

Variable	Categories	AH	Non-AH	*p*-Value	Test Statistics	Effect Size
Encephalopathy	Yes	20 (20.0)	15 (16.3)	0.5	0.439	0.048
	No	80 (80.0)	77 (83.7)			
Cirrhosis	Yes	34 (34.0)	12 (13.0)	**<0.001**	11.551	0.245
	No	66 (66.0)	80 (87.0)			
Ascites	Yes	42 (42.0)	10 (10.9)	**<0.001**	23.514	0.350
	No	58 (58.0)	82 (89.1)			
Underlying liver disease	Yes	84 (84.0)	51 (55.4)	**<0.001**	18.731	0.312
	No	16 (16.0)	41 (44.6)			
Taking hepatotoxic medications	Yes	24 (24.0)	34 (37.0)	0.05	3.815	0.141
	No	76 (76.0)	58 (63.0)			
Hepatitis panel	Positive	3 (3.0)	15 (16.3)	**0.002**	9.983	0.228
	Negative	97 (97.0)	77 (83.7)			
MELD-Na score at admission (Mean ± SD)	–	20.9 ± 8.7	13.9 ± 7.4	**<0.001**	4.972	0.849
Maddrey’s discriminant function score at admission (Mean ± SD)	–	22.1 ± 8.58	N/A *	N/A	N/A	N/A
Hypertension	Yes	44 (44.0)	53 (57.6)	0.06	3.550	0.136
	No	56 (56.0)	39 (42.4)			
Hyperlipidemia	Yes	27 (27.0)	31 (33.7)	0.3	1.019	0.073
	No	73 (73.0)	61 (66.3)			
HIV	Yes	1 (1.0)	4 (4.3)	0.2	2.117	0.105
	No	99 (99.0)	88 (95.7)			
Treatment with glucocorticoids	Yes	19 (19.0)	9 (9.8)	0.07	3.268	0.130
	No	81 (81.0)	83 (90.2)			

Notes: * Maddrey’s discriminant function score was applicable only to those with AH and thus were not included in the analysis for non-AH patients. *p* values < 0.05 are considered statistically significant and are bolded in the table. Data are represented as frequencies and proportions unless stated otherwise. Some categories may not add to 100% due to missing data.

**Table 3:** Bivariate comparisons of the hospital outcomes (N = 192).

Variable	Categories	AH	Non-AH	*p*-Value	Test Statistics	Effect Size
Disposition	Home	61 (61.0)	59 (64.1)	0.9	0.604	0.056
	Facilities	13 (13.0)	11 (12.0)			
	Death	8 (8.0)	5 (5.4)			
	AMA	6 (6.0)	6 (6.5)			
	Others	12 (12.0)	11 (12.0)			
Length of hospital stay (Mean ± SD)	-	6.25 ± 1.18	6.91 ± 1.46	0.4	−0.804	−0.116

Other category includes police custody, shelter, group home, and hotel.

**Table 4:** Predictors or risk factors of AH (Multivariate Logistic Regression).

Variable(s)	Odds Ratios Estimate	95% Confidence Limits		*p*-Value
Age	0.985	0.956	1.015	0.33
Gender	0.594	0.276	1.276	0.18
Race, White vs. Non-Hispanic Black	1.406	0.519	3.806	0.50
Hispanic vs. Non-Hispanic Black	0.867	0.225	3.338	0.84
Other race vs. Non-Hispanic Black	0.076	0.005	1.092	0.06
Insurance, Public vs. uninsured	0.352	0.117	1.062	0.06
Insurance, Private vs. uninsured	0.517	0.15	1.788	0.30
Body mass index	0.999	0.949	1.052	0.96
Prior history of Alcohol Hepatitis (Yes vs. No)	1.539	0.608	3.898	0.36
Binge drinking (Yes vs. No)	**2.698**	1.079	6.745	**0.03**
Heavy drinking (Yes vs. No)	**3.169**	1.348	7.452	**0.01**
Hypertension (Yes vs. No)	0.56	0.256	1.224	0.15
Hyperlipidemia (Yes vs. No)	0.873	0.38	2.007	0.75
Underlying liver disease (Yes vs. No)	2.026	0.81	5.067	0.13
Cirrhosis (Yes vs. No)	**3.392**	1.306	8.811	**0.01**

**Table 5:** Predictors or Risk Factors of Inpatient Mortality (Logistic Regression).

Variable(s)	Odds Ratios Estimate	95% Confidence Limits		*p*-Value
Age	0.979	0.913	1.05	0.54
Gender	0.412	0.088	1.80	0.24
Race, White vs. Non-White	2.72	0.492	22.3	0.29
Probable AH vs. Possible AH	**6.79**	1.38	44.9	**0.03**
Insurance, Public vs. uninsured	0.815	0.113	7.39	0.84
Insurance, Private vs. uninsured	2.73	0.324	28.8	0.36
Body mass index	0.988	0.893	1.08	0.8
Prior history of Alcohol Hepatitis (Recurrent AH vs. First Time AH)	2.44	0.574	10.9	0.23
Binge drinking (Yes vs. No)	0.515	0.094	2.72	0.43
Heavy drinking (Yes vs. No)	0.665	0.077	5.46	0.7
Hypertension (Yes vs. No)	6.51	0.949	35.7	**0.02**
Hyperlipidemia (Yes vs. No)	0.189	0.021	1.05	0.08
Cirrhosis (Yes vs. No)	1.18	0.262	5.19	0.83

## Data Availability

The data presented in this study are available on request from the corresponding author. The data are not publicly available due to HIPAA.
